# Errata to “A revised taxonomy of the ammonoid genus *Desmoceras* from Japan and southern Sakhalin” [Proc. Jpn. Acad., Ser. B 80, No. 5, 225–229 (2004)]

**Published:** 2004-06-01

**Authors:** Tatsuro Matsumoto, Tamio Nishida

In this paper, the phrases should be corrected as follows:

(page 226, right column, line 11)

For “**4. *Desmoceras (D.) japonicum*** Yabe.^1)^”

read “**4. *Desmoceras (P.) japonicum*** Yabe.^1)^”.

(page 229, left column, line 12)

For “**5. *Desmoceras ezoanum*** Matsumoto.”

read “**5. *Desmoceras (P.) ezoanum*** Matsumoto.”.

